# Distal Duodenogastrostomy or Proximal Jejunogastrostomy in the Management of Ultra-Short Bowel

**DOI:** 10.1007/s11605-017-3654-0

**Published:** 2017-12-22

**Authors:** Tjipke Olivier Hofker, Mirjam Anna Kaijser, Vincent B. Nieuwenhuijs, Johan Frederick Michel Lange, Hendrik Sijbrand Hofker

**Affiliations:** 1 Department of Surgery, University Medical Center Groningen, Hanzeplein 1, 9713 GZ Groningen, the Netherlands; 2 Department of Surgery, Isala Hospital, Zwolle, the Netherlands

**Keywords:** Duodenogastrostomy, Proximal jejunogastrostomy, Short bowel syndrome, Near total enterectomy, Total parenteral nutrition

## Abstract

Inflammatory bowel disease, vascular disease, volvulus, adhesions, or abdominal trauma may necessitate extensive small-bowel resection resulting in an ultra-short distal duodenal or jejunal stump. If this distal duodenal or short jejunal stump is too short for stoma creation and bowel continuity restoration is hazardous or not possible at all, a distal duodenogastrostomy or proximal jejunogastrostomy in combination with drainage of the stomach is an option to prevent stump leakage. Although successful, this distal duodenogastrostomy has been described only in very few patients and in older records. We reintroduced this technique and describe a recent series of patients that confirms its usefulness in certain conditions. The technique of the distal duodenogastrostomy or proximal jejunogastrostomy with gastric drainage was used for the management of the difficult distal duodenum stump in five critically ill patients undergoing extensive bowel resection. Four patients with small-bowel ischemia and one patient suffering from perforating Crohn’s disease and small-bowel volvulus were treated  successfully. The gastrostomies were subsequently converted to a duodenotransversostomy (in two patients) or the patients underwent small-bowel transplantation (two patients). One patient still has a jejunogastrostomy just after the duodenal-jejunal transition. In all five patients, the distal duodenogastrostomy or proximal jejunogastrostomy in combination with gastric drainage functioned well up to restoration of bowel continuity. In one patient, distal duodenogastrostomy and transabdominal gastric drainage functioned well for 5 years. No anastomotic leakage occurred. This procedure provides a feasible solution for an ultra-short bowel at emergency laparotomy. It enhances the surgical armamentarium and provides treatment options for these patients that were perhaps previously deemed unsalvageable.

## Introduction

When ischemic bowel disease, severe inflammatory bowel disease, volvulus, adhesions, or abdominal trauma may necessitate extensive small-bowel resection or even near total enterectomy, surgeons may be confronted with a short distal duodenal stump or ultra-short proximal jejunal stump. These stumps are threatened by accumulating volumes of gastric contents, pancreatic juices, and bile, all contributing to a high risk of stump leakage. Stump leakage will lead to abdominal sepsis in already critically ill patients. The finding of ischemic bowel up to the level of the duodenojejunal junction can therefore be a reason for surgeons to refrain from further curative treatment.

Little has been published about how to manage or prevent the leakage of the distal duodenal stump. If stoma creation of the stump is achievable, this is a very safe option. Nauta showed that the duodenum itself can also be anastomosed to small bowel with a duodenojejunostomy at the descending duodenum.^1^ If feasible, an anastomosis between the duodenal stump and colon can be made in cases in which after near total enterectomy the colon is still well vascularized.^2^ However in severe cases with more extensive or even near total enterectomy, the duodenal stump may be too short for external stoma creation of too poor condition to restore bowel continuity or there may be too little bowel available to restore continuity at all. In these cases, prevention of blow out of the stump is crucial.

More literature is available discussing the prevention and treatment of leakage of the proximal duodenal stump, after for instance distal gastric resection in oncologic and bariatric surgery.^3^–^7^ As the two problems occur in the same organ in a similar cascade, accumulation of digestive fluids leading to a blow out of the weakest point, lessons can be learned from these publications. Schein states that the best way to avoid a difficult duodenal stump and its potentially lethal complications is not to create a stump at all: by either avoiding a gastrectomy or, if forced to do one, restoring continuity with a gastroduodenostomy.^8^

In case of the distal duodenal stump, the options of restoring continuity may be not easily available. If an intra-abdominal distal duodenal stump is inevitable, options to prevent stump leakage are to insert a transduodenal drain, a drain in duodenum stump, or to drain the duodenum by means of a nasogastric drain that’s advanced into the duodenum. Rates of success of these approaches are unknown.

The technique dealing with the duodenal stump that we report on was first mentioned in case reports by Langer in 1973 and 2 years later by Bordos.^9^,^10^ In those two articles, patients were described that required total parenteral nutrition (TPN) for short-bowel syndrome. In both series, one patient suffered from duodenal and jejunal stump fistulas. The stump fistulas in both patients were managed successfully by anastomosing them to the stomach, which was drained externally. Bosscha and van Vroonhoven described the duodenogastrostomy technique in more detail.^11^ In their series, it was used as a temporary measure to divert the fecal stream from enterocutaneous fistulas in four patients with nearly inaccessible abdomen. In none of the in total six described catabolic patients, anastomotic leakages of the duodenogastrostomies were reported.

We adopted this technique for patients with an extensive small-bowel resection in order to manage a distal duodenal or proximal stump fistula or to prevent stump leakage. Our experience with the duodenogastrostomy and jejunogastrostomy is described and recommendations for their use are proposed.

## Methods

A retrospective study of our patient database in the period 2000–2015 was performed. All patients, in whom a distal duodenogastrostomy or proximal jejunogastrostomy were intended, were included in the study. A descriptive analysis of the cases is presented.

### Operative Technique

**Fig. 1:**
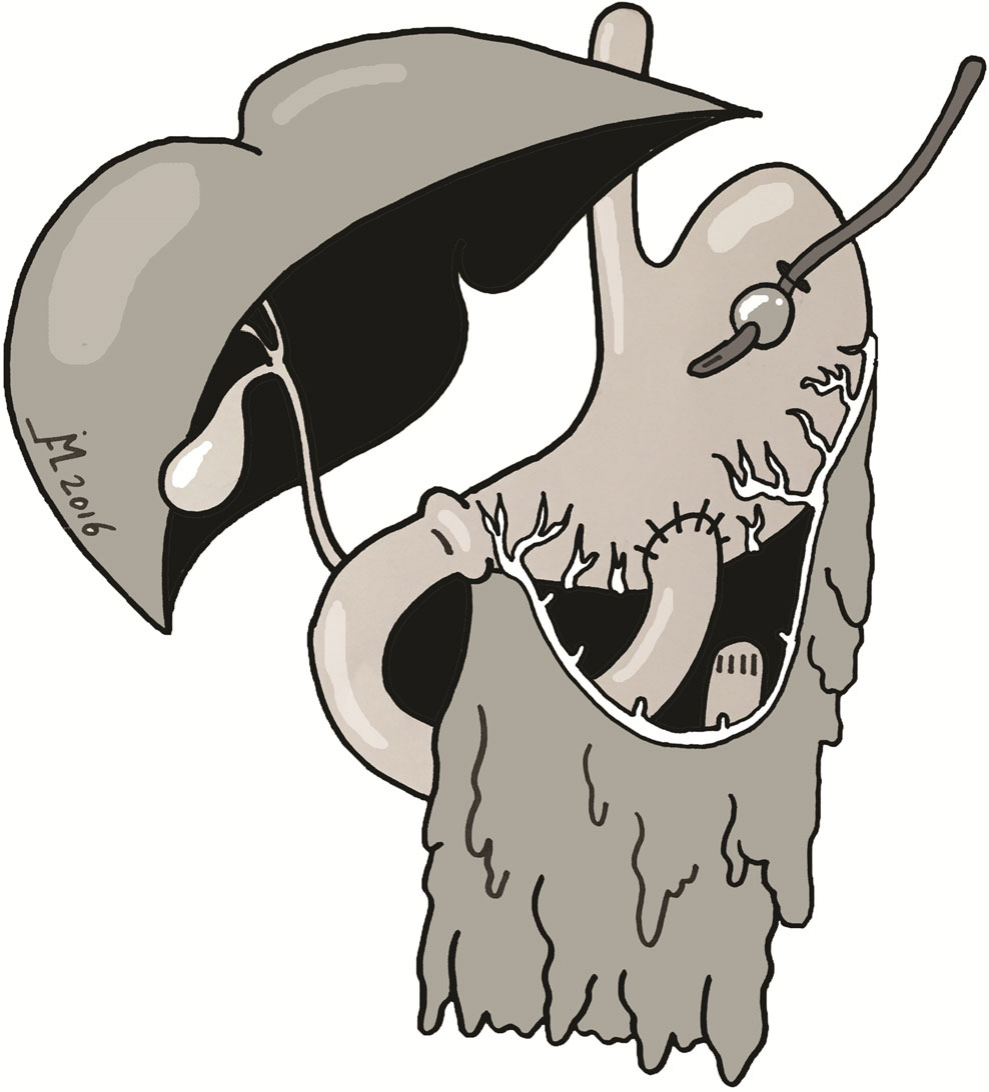
Schematic display of duodenogastrostomy and percutaneous tube gastrostomy

After resection of all irreversible injured small bowel and colon, in each case, options for anastomosis or ostomies were inventoried. If restoration of bowel continuity was technically feasible and conditions for healing of the anastomosis were favorable, this was the first choice of treatment. Conditions considered to preclude bowel continuity restoration were compromised vascularity, unstable hemodynamics, or otherwise compromised healing of anastomoses. If patients did not meet the requirements to perform a safe anastomosis, the second option was to create an end ostomy of small bowel. If due to the length of the remaining bowel such a distal duodenostomy was not an option, we decided to evaluate the local situation for a distal duodenogastrostomy or proximal jejunogastrostomy combined with gastric drainage as an alternative for direct external duodenal tube drainage. As Bosscha and van Vroonhoven already noticed, the omental bursa often remains accessible even after multiple re-operations.^11^ To create the duodenogastrostomy, if the transverse colon is still in situ, the lesser sac can be opened through the avascular part of the gastrocolic ligament. The transverse mesocolon is incised left to the aorta and the ascending duodenum is mobilized. The duodenum or proximal jejunal stump is then pulled upward to the antrum of the stomach and a hand-sewn end-to-side duodenogastrostomy or jejunogastrostomy is performed at the anterior or posterior site of the greater curvature. Lysis of adhesions between the posterior side of the stomach and the peritoneal coverage of the posterior part of the lesser sac can help to pull the antrum inferiorly over the distal duodenum. Typically, the distal duodenum is anastomosed in an end-to-side fashion to the posterior part of the antrum. Also, the duodenum can be mobilized posteriorly and retracted underneath the mesenteric superior artery to perform a side-to-side fashioned duodenogastrostomy. If the distal duodenum or proximal jejunum is long enough and the posterior stomach wall has firm adhesions to the peritoneum covering the pancreas, an anterior side-to-side or end-to-side distal duodenogastrostomy or jejunogastrostomy may be created. Either a nasogastric tube or a passive draining anterior tube gastrostomy is added to divert the gastric and duodenal fluid to prevent retention (Fig. 1). An abdominal drain is placed near the anastomosis to allow early detection of anastomotic leakage.

Reversal of the duodenogastrostomy or proximal jejunogastrostomy to restore bowel continuity is performed by mobilizing the anastomotic area, then detaching the anastomosis, closing the hole in the stomach and anastomosing the duodenum or jejunum to the other part of the bowel. No special workup was done before reversing this anastomosis.

## Results

The first patient is a 46-year-old woman diagnosed with acute thrombosis in the celiac trunk and the superior mesenteric artery (SMA). A resection of the small bowel from 10 cm distal to Treitz ligament and a right hemicolectomy were performed. At that time, she was considered not fit to restore bowel continuity by the referring hospital. After consultation of our departments’ staff, the referring hospital performed a transmesocolic end-to-side sutured single-layer jejunogastrostomy at the posterior part of the antrum to prevent a blow out of the distal duodenal stump. She received a nasogastric tube to prevent gastric retention, later replaced by a percutaneous tube gastrostomy. Thrombophilia screening and vascular workup did not reveal any underlying disorder. Three months later after recovery from the severe abdominal sepsis, the duodenogastrostomy was converted to a jejunotransversostomy. Because of the short bowel, she remained TPN dependent. Fluid balance was maintained by adding 1 l of sodium chloride solution every other day. A proton pump inhibitor was infused daily. Three years later, she successfully underwent small-bowel transplantation.

The second patient is a 20-year-old woman diagnosed with Crohn’s disease. One year after an uncomplicated subtotal colectomy, she suffered from torsion of the small bowel around the ileostomy, leading to ischemia and subsequent resection of almost the entire small bowel, leaving only an ultra-short distal duodenal stump. She was referred to our center with a severe abdominal sepsis caused by distal duodenal stump leakage. At referral, two Foley catheters in the duodenal stump and a vacuum-abdominal-closing system inadequately controlled the leakage. Apart from leakage of duodenal contents alongside the Foley drain, these contents also flowed into her abdomen through two duodenal perforations or fistulas, causing ongoing peritonitis. At laparotomy, the adhesive duodenum was mobilized posteriorly. Two Crohn’s fistulas in the descending duodenum were oversewn. The mobilized duodenal stump was sutured single layer end-to-side to the posterior aspect of the distal antrum of the stomach. A tube gastrostomy was placed anteriorly and the patient was put on chronic TPN. Acute renal failure due to the abdominal sepsis completely resolved. Five years later, she too was treated successfully with a small-bowel transplantation to enhance quality of life. This patient was treated for the longest period of time by means of the distal duodenogastrostomy and transabdominal gastric drainage. She was happy to be able to drink as a social skill but she disliked the fact that the liquid immediately ran into a plastic draining bag that she carried in her handbag. Also, the inability to eat decreased her quality of life significantly. Remarkably, the patient did not show a high gastrostomy output. Half a year after the duodenogastrostomy, she required only 1 l of normal saline fluid in addition to 2 l of TPN, and 1 year after the near total enterectomy, this was no longer needed as a standard supplement. A proton pump inhibitor was infused daily; she did not require a somatostatin analogue.

The third patient is a 22-year-old male diagnosed with thrombosis of the SMA leading to ischemia of the small intestine. A resection was performed of almost the complete small bowel and right colon. An anastomosis between the duodenum and transverse colon was deemed unsafe. The ascending duodenum was perfused sufficiently. A window was created in the transverse mesocolon and an end-to-side sutured single-layer duodenogastrostomy at the posterior part of the antrum was performed, combined with an anterior tube gastrostomy. This gastrostomy initially produced 4.5 l a day; within 1 year, this had diminished to 1 l. He did not require a proton pump inhibitor or intravenous fluid in addition to the 2 l of TPN. Screening for thrombophilia was negative. Several months after this procedure, the occluded SMA was revascularized by endovascular stent placement. One year later, the duodenogastrostomy was converted to a duodenotransversostomy and the percutaneous gastric tube was removed. Currently, he is screened for small-bowel transplantation.

The fourth patient is a 53-year-old female diagnosed with mesenteric thrombosis. After consultation by the referring hospital of our departments’ staff, the referring hospital performed a resection removing the small bowel and right hemicolon. The stomach, duodenum, and the proximal 10 cm of the jejunum were vital. The jejunum was mobilized and attached to the posterior side of the stomach with an end-to-side hand-sewn jejunogastrostomy. A Foley catheter was used as a tube gastrostomy. A central venous catheter was inserted for parenteral nutrition. Screening for thrombophilia was negative. Apart from TPN, 1 year after the operation, she required 1 l of saline and a proton pump inhibitor a day. Currently, she is screened for small-bowel transplantation as well.

**Fig. 2:**
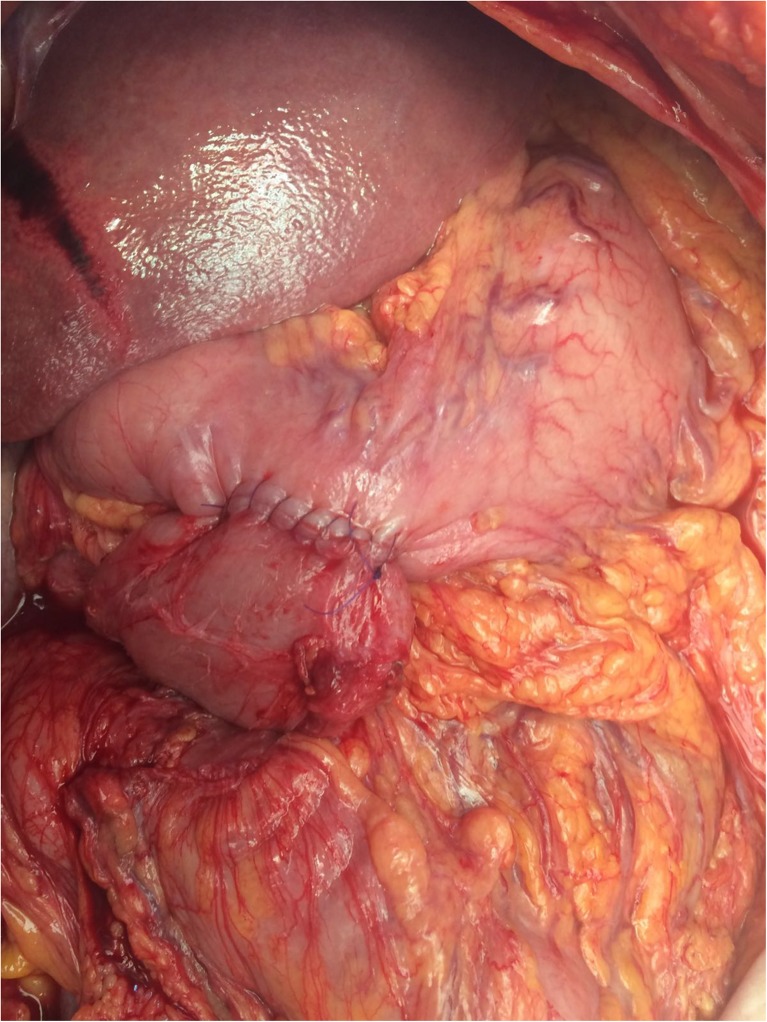
Creation of a hand-sewn side-to-side duodenogastrostomy at the anterior part of the antrum in one patient

The fifth patient is a 60-year-old female with mixed connective tissue disease. After recurrent episodes of self-limiting abdominal complaints in the past years, she was hospitalized with abdominal pain rapidly evolving into abdominal sepsis. Abdominal CT revealed a severely distended small intestine. At laparotomy, a completely necrotic, but non-perforated, small bowel was observed, due to a 360-degree mesenteric torsion. The small intestine was rotated back into the normal position, but the ischemia was irreversible. Transection of the gut was performed at the ligament of Treitz and ascending colon. The entire small intestine was thus resected. After hemodynamic stabilization in the intensive care unit, a second look the next day showed no further ischemia. The duodenum was mobilized to ease retraction lateral to the superior mesenteric artery. The omental bursa was opened both through the gastrocolic ligament and through the transverse mesocolon. Next, the distal duodenum was anastomosed side-to-side to the anterior portion of the antrum of the stomach, with a single-layer hand-sewn technique (Fig. 2). Finally, a Foley catheter was used as a tube gastrostomy. She recovered from the abdominal sepsis and TPN was administered. In the postoperative course, a deep venous thrombosis occurred that was treated with anticoagulants without further complications. After the operation, she required 2 l of saline a day, as well as a proton pump inhibitor and a somatostatin analogue. Currently, she is recovering at home.

### Early Complications

In the postoperative courses of the patients after the duodenogastrostomy or proximal jejunogastrostomy, no re-operations were needed. Anastomotic leakage or mortality did not occur. Review of the patient records revealed two sepsis-related complications, acute renal failure, and deep venous thrombosis, both completely resolved. Wound complications or other postoperative infections were not described.

### Long-term Results

For long-term follow-up of the nutritional status and TPN-related complications, a specialized team followed the patients, either in our hospital or in a specialized TPN center. All patients had recurrent complications of the central venous catheter, either as thrombosis or as infections, requiring changes of the catheters, without further morbidity. Liver-related complications due to TPN were treated by changing the composition of the TPN; none of the patients suffered from liver failure. Depending on the needs of the patients, different formulas of TPN were given including Clinimix® N17G35E, Olimel®, and Smovkabiven®, if necessary combined with Omegaven®, Addamel®, and Cernevit®. As described above, the fluid balance was maintained by administering 0.9% sodium chloride intravenous, with a maximum of 2 l a day. Administering 40–80 mg proton pump inhibitors downregulated gastric as well as digestive fluid productions. Only the fifth patient has a somatostatin analogue administered.

All five patients tolerated the situation including the continuous transabdominal gastric drainage. In two patients, bowel continuity was restored both for medical reasons and to improve quality of life. The two patients in which bowel continuity with autologous bowel was not possible opted for bowel transplantation. One of them has been transplanted. Another patient also chose bowel transplantation for quality of life reasons after successful bowel continuity restoration.

## Discussion

A distal duodenogastrostomy or proximal jejunogastrostomy in combination with gastric drainage is a feasible and safe option to prevent distal duodenal stump leakage after enterectomy for various indications. In ultra-short bowel patients, it enhances the surgical armamentarium and it provides treatment options for these patients that were perhaps previously deemed unsalvageable. It can even be applied in septic, unstable patients with an already leaking duodenal stump. It may be used as a step up to restore bowel continuity or small-bowel transplantation.

Preferably, a duodenal stump or very short jejunal remnant should be anastomosed to other bowel to restore continuity. The bailout, if no bowel is available for re-anastomosis or if the condition of the patient or bowel is considered too poor (sepsis, ischemia, malnourishment), is to exteriorize the bowel as a duodenostomy. However, if the stump is too short for exteriorization, accumulating volumes of gastric contents, pancreatic juices, and bile contribute to a high risk of stump leakage, comparable to the proximal duodenal stump leakages.^7^ In these cases, the standard of care to prevent stump leakage is to drain the duodenum by luminal drains inserted through the duodenal wall, through the duodenal stump or by means of a gastric drain that’s advanced through the pylorus into the duodenum. A problem that may be encountered in the transduodenal and duodenal stump drains is leakage alongside the drains into the abdominal cavity, if no direct contact between the duodenum and the abdominal wall is present. This was also the case in the second patient in our series. The risk of a duodenal drain advanced through the stomach is dislocation and consequently inadequate drainage. Because of its tube length and limited diameter, it’s also at risk for insufficient drainage and continuous suction should perhaps be applied.

The distal duodenal or short proximal jejunal stump can also be anastomosed in an end-to-side or side-to-side fashion to the stomach and include nasogastric or transabdominal drainage of the stomach. The feasibility of this technique for the indication of short bowel and the presence of leaking duodenal or short jejunal stump has been shown by Langer et al. and Bordos et al.^9^,^10^ The authors describe a patient who respectively suffered from a leaking duodenal stump and a leaking short jejunal stump. These patients were mentioned among other cases in articles that focused on TPN. Langer pioneered in the use of intravenous alimentation in two patients, the second of whom underwent a duodenogastrostomy with a gastric tube, combined with a vagotomy to reduce the gastric juice production, after severe intestinal fistulae occurred after ischemia of the small bowel. Bordos’ patient underwent a jejunogastrostomy after a prolonged period of abdominal sepsis by enteric leakage. Combined with gastric drainage, the distal transbursal end-to-side duodenogastrostomy was also described in more detail by Bosscha and van Vroonhoven to treat enteric fistulas in four patients. After closure of these fistulas, the duodenum was reconnected to the jejunum.^11^ No leakage was seen of the duodenogastrostomies in their series of catabolic patients.

For those reasons, especially high risk of duodenal stump leakage and safety of duodenogastrostomy anastomosis, we started to use this technique to prevent anastomotic leakage in hazardous restorations of proximal bowel continuity, to prevent duodenal stump leakage and to treat distal duodenal stump leakage. In both cases in which a duodenotransversostomy was technically possible, it was judged preoperatively to be unsafe. In the patient with a leaking duodenal stump, the duodenum had to be mobilized to allow the creation of the distal duodenogastrostomy. No anastomotic leakage of the gastrostomies occurred. Because of extensive resections and severe sepsis, the distal duodenogastrostomy or proximal jejunogastrostomy in our series was maintained longer (up to 5 years) than in the Bosscha series (2 to 5.5 months). The situation could also be maintained for such a long time interval because of the absence of severe complications. Furthermore, the technique had the desired effect of avoiding and treating of leakage. In three cases, the procedure was followed by either restoration of continuity with or without small-bowel transplantation. In one patient with acute thrombosis of the SMA, the occlusion of the SMA was treated by endovascular techniques before restoration of bowel continuity.

All patients tolerated the combination of a duodenogastrostomy or proximal jejunogastrostomy and external gastric drainage well. None of the patients had an additional vagotomy like Langer performed in his patient.^9^ We hypothesized that the very proximal gastro-enteric drainage with a percutaneous gastrostomy would result in large output volumes. This would implicate the need for large amounts of intravenous fluid supplements. Remarkably though, after the recovery period, only 0 to 2 l of saline were needed to compensate for the intestinal losses. As only the fifth patient, with the shortest follow-up, was administered a somatostatin analogue, with other patients only requiring a proton pump inhibitor, apparently fluid production by stomach, pancreas, and liver was downregulated by the non-physiologic anatomy. Potential high enteric losses therefore should not be a reason to refrain from this technique.

A major advantage of this technique is that it seems to be possible to connect the distal duodenal stump to the stomach, even in acute situations with bowel ischemia, presence of a leaking duodenal stump, sepsis, and Crohn’s disease.

Obvious limitations of this study are the retrospective nature and the limited number of cases. However, as this problem is rare, comparable numbers of patients are described in earlier case series. Assumptions about comparisons with proximal duodenal stump leakage have been made.

## Conclusion

Our experience with the distal duodenogastrostomy and gastric drainage is positive. It created a safe and reversible anastomosis in very sick patients. The distal duodenogastrostomy and proximal jejunogastrostomy provide a good alternative to a duodenocolostomy or a drain in the duodenal stump. These gastrostomies are a safe bridge to a permanent solution (restoration of bowel continuity or small-bowel transplantation). We believe that surgeons dealing with these kind of abdominal catastrophes should be aware of this technique.
